# Rulers of engagement: A novel approach to measuring engagement in a large Mental and Behavioral Health Institute

**DOI:** 10.1017/cts.2025.10145

**Published:** 2025-09-02

**Authors:** Bridget N. Murphy, Stephanie Weber, Jill Cordonnier, Monica J. Mitchell

**Affiliations:** 1 Division of Behavioral Medicine and Clinical Psychology, Cincinnati Children’s Hospital Medical Center, Cincinnati, OH, USA; 2 Division of Developmental and Behavioral Pediatrics, Cincinnati Children’s Hospital Medical Center, Cincinnati, OH, USA; 3 Department of Pediatrics, University of Cincinnati College of Medicine, Cincinnati, OH, USA; 4 Cincinnati Children’s Hospital Medical Center Mental and Behavioral Health Institute, Cincinnati, OH, USA

**Keywords:** Community engagement, measurement, mental health, behavioral health, evaluation

## Abstract

While community engagement (CE) has been increasing in the mental and behavioral health fields, evaluation of CE remains a challenge. Currently, there are no published evaluation tools that assess frequency of engagement, and many CE measures are not based on established engagement theories. Based on the International Association for Public Participation’s CE continuum, the CE team of the Mental and Behavioral Health Institute (MBHI) at a large pediatric hospital developed a system of measurement to describe frequency of engagement across eight initiatives. This tool, the Frequency of Active Community Engagement (FACE) measure, was administered to the leaders of each of the participating MBHI teams. FACE summarized CE frequencies for three target populations (youth, caregivers, and community members) for each team. Follow-up team meetings provided additional descriptive information for the development of CE goals. In this special communication, we describe this data collection approach, CE results, as well as future directions and potential uses for FACE as an evaluation tool.

## Introduction

Community engagement (CE) is increasingly being recognized as a way to increase the rigor, reach, and relevance of both research and clinical services [1]. CE is foundational to successful translation of services to communities, as engagement can foster trust, enhance the relevance and interpretation of research data, and allow community and academic partners to anticipate and address barriers to implementation [2]. The goal of genuine CE is to ensure transparency when discussing roles, responsibilities, goals, and expected outcomes of the partnership. A challenge for academic and community partnerships is to balance power in the relationship to achieve bi-directional benefits, while highlighting complementary skills and resources [3]. For example, if community and academic stakeholders are working together on an asthma intervention grant, roles may vary depending on the goals of the initiative (e.g., parents of children with asthma may provide consultation to a pediatric care improvement initiative). To successfully work within these nuances, partners must be able to define and measure their engagement work.

Currently, there are a variety of ways to measure CE, including measuring how engagement happens (process), the circumstances around the engagement (context), and the outcomes of the engagement (outcomes) [4]. A review of community engagement measures by Luger *et al* [4]. found 69 measures of context, process, and outcomes used between 2009 and 2018. Though the measures identified were potentially generalizable beyond the studies for which they were used, the authors concluded that there is a need for consistency in measurement across different projects and institutions. Furthermore, another review of CE measures by Bolvin *et al* [5]. found that of the 27 measures included, only 11% were based on literature review and only 7% were tested for psychometric properties. These findings highlight the limited psychometric rigor of CE measures and the need for measures that can be used over time and across different projects.

Measuring CE descriptively – such as tracking frequency and level of CE – often occurs in the context of measuring the quality of partnerships or their impact on health outcomes. The value of tracking frequency is to measure how often various types of CE activities occur and with whom (e.g., youth and patients, parents and caregivers, respective community), as well as to identify gaps in representation [6]. This approach fosters the inclusion of community members or increases in CE based on the needs and goals of the work. Using descriptive levels of engagement and participation to engage partners is novel to CE evaluation efforts and supports tracking of partner engagement efforts over time.

### CCHMC mental and behavioral health institute

Cincinnati Children’s Hospital Medical Center (CCHMC) is a nonprofit, comprehensive pediatric health system internationally recognized as a leader in research, education, patient care, advocacy, and innovation. In 2023, CCHMC inaugurated the Mental and Behavioral Health Institute (MBHI), which brings the departments of Behavioral Medicine and Clinical Psychology (psychology), Child and Adolescent Psychiatry (psychiatry), and Developmental and Behavioral Pediatrics (developmental pediatrics) in alignment under one institute (www.cincinnatichildrens.org/services/m/mental-behavioral-health). Cross-service collaboration can help providers address complex problems in a comprehensive manner, assist patients with navigating a complicated system more effectively, and enhance accessibility to services, especially with under-resourced populations [7].

The MBHI has identified eight top priority initiatives to advance clinical care, research, professional education, and access for mental and behavioral health services. These priorities are designed to improve mental health access across the care continuum by addressing both upstream prevention efforts and downstream clinical care outcomes. These initiatives include: (i) implementation of exposure coaching to expand on our clinical treatment options for anxiety disorders; (ii) development of specialized care pathways for common mental health disorders (e.g., anxiety, depression) to ensure a continuum of care for varying levels of patient needs; (iii) expansion of an integrated behavioral health program to increase early intervention and the number of patients who receive mental health care in their medical home; (iv) development of an evidence-based certification program to increase the knowledge and confidence of the community mental health provider workforce; (v) increased spread of the Pediatric Improvement Network for Quality (PINQ) learning network to engage more community providers in evidence-based mental health care; (vi) increased course offerings of the Project Extension for Community Healthcare Outcomes (ECHO™) training program for community-based providers; (vii) implementing the Zero Suicide framework to systematically reduce the number of youth suicide deaths in our region; and (viii) creating a family and system navigation network to remove barriers to accessing mental health care.

In addition to prioritizing the above-mentioned goals, MBHI leadership established an expectation that each initiative team engages patient, caregiver, and/or community voices in their work. As such, the MBHI CE team was assembled to develop a framework and system of measurement and assist teams in setting and achieving CE goals. The MBHI CE team is led by two psychologists with expertise in CE activities related to healthcare access, community building, and advocacy. A postdoctoral fellow in psychology provides research and overall team support. A program manager provides specific administrative support to the MBHI CE team and collaborates with the institute’s other program manager to ensure consistent communication, timely goal-setting, and programmatic efficiency. This team also works in conjunction with the institution’s Center for Clinical and Translational Science and Training (CCTST), which facilitates academic and community partnerships for the hospital and the Office of Community Relations.

### Current paper

The current paper describes the measure developed by the MBHI CE team as well as the baseline data collected. The process-oriented measure focuses on descriptive, categorical data, namely the frequency and type of CE activities, with the overarching goal of shifting the paradigm within the MBHI to emphasize lived experience and promote co-production across programs and initiatives. Examples of goals established by the MBHI initiative teams based on the initial data are discussed.

## Methods

### Survey development

**Figure 1.**
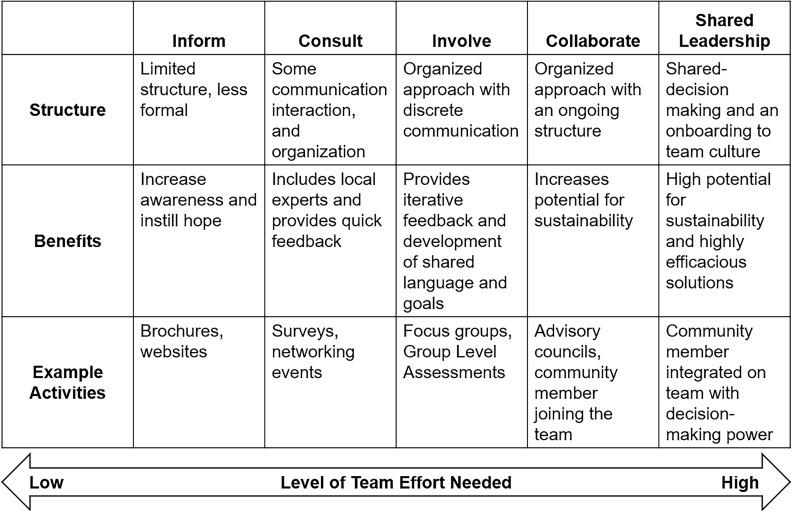
Continuum of Community Engagement (CE). *Note.* This continuum was published by Mitchell, Reily, and Crosby 9 and based on the International Association for Public Participation’s definition of levels of Community Engagement 8. The Frequency of Active Community Engagement (FACE) tool asked respondents to rate the frequency of engagement at each level.

The Frequency of Active Community Engagement (FACE) tool, the measure the MBHI CE team created, was based on the International Association’s for Public Participation (IAP [2]) continuum [8]. and Mitchell et al’s [9] published model. This model includes a continuum of five levels of CE: Inform, Consult, Involve, Collaborate, and Shared Leadership. The level of engagement is based on both the engagement activities and who holds the decision-making power. At the *Inform* level, academic partners provide information to the community and coordinate outreach. For *Consult*, academic partners solicit feedback, ideas, and information from the community. At the *Involve* level, community partners are more active in generating ideas, and bidirectional communication and cooperation are established. At the first three levels, the academic partner holds the decision-making power. At the *Collaborate* level, community partners are typically engaged with the project or initiative from the inception through completion, and decision-making becomes more balanced between the academic and community partners. *Shared leadership* is the highest level of engagement, and it involves community partners having equal power in the collaboration and influence in the final decision-making. See Figure 1.

Using these levels of CE as the descriptive foundation for the measure, the MBHI CE team opted to focus on measuring the frequency of engagement at each level with three populations of interest: youth and patients, caregivers, and community members. The rationale for this approach was that each initiative would have a different optimum level of CE which may differ based upon population. While some may be appropriate for shared leadership, other initiatives would be a better fit for lower levels of engagement. It was expected that initiatives may engage at multiple levels simultaneously, and it was necessary for the measure to be able to reflect different activities while not penalizing initiatives that were not appropriate for shared leadership. Given the MBHI is a new endeavor, it was important for the CE team to gather a concrete appreciation of the volume of CE activities in current practice. This process offered the CE team opportunities to share the Institute leaders’ message on the importance of inclusion of community members in the initiatives while also building rapport with project and team leaders. Frequency of activities provided respondents with a simple and quick experience which allowed the CE team to gather preliminary and pilot information. The full survey can be found in Supplementary Materials.

### Survey administration and follow-up

The MBHI CE team administered the survey through Microsoft Forms and encouraged several key leaders from each initiative to complete it. MBHI teams were emailed the survey link and a detailed description of the timeline and expectations. In total, 52 team members were asked to complete the survey. Participants were asked to think about their team’s work to date on the initiative of interest and rate their perception of the level of engagement for three distinct populations: patients/youth, caregivers/families, and community members/community leaders. The survey included definitions for each of these groups. For example, patients would be considered those seen for clinical services in the hospital system, whereas youth might be students in local schools, part of youth groups, or affiliated with community programs. Caregivers were those who have children seen at CCHMC, and families were adults with youth in the community who are not necessarily patients at CCHMC, such as parents of children at a specific school or church. Examples of community members/community leaders were also shared and include staff working in schools and community mental health agencies, as well as community residents.

Participants rated each level of engagement based on the frequency of engagement at that level with the following options: not engaged, previously engaged but not currently, engaged 1–3 times per year, engaged quarterly, engaged monthly, and engaged multiple times per month. Results were calculated based on the frequency of engagement, and the data were considered categorical and descriptive. The categories of not engaged and previously but not currently engaged were scored as zero. Engaged 1–3 times per year was given one point, engaged quarterly was two points, engaged monthly was three points, and engaged multiple times per month was awarded four points. The response totals were aggregated by dividing the total points by the number of respondents within the initiative group to produce an average level of engagement reported across respondents within an initiative. Results were calculated for each individual initiative and compiled for the MBHI as a whole to illustrate frequencies of engagement based on level and across populations.

The MBHI CE team sent a request for survey completion two weeks prior to a scheduled virtual meeting with that initiative’s team. This approach provided a short window for survey completion with a clear endpoint and expectations that results would be discussed at the meeting. These follow-up meetings provided opportunities for the CE team to describe our overall mission of increasing CE across the MBHI, which facilitated consensus-building of the importance of elevating the voice of those with lived experience. Additionally, these conversations allowed team members to discuss their ratings, clarify discrepancies, and confirm alignments. The meetings also provided space for the CE team to assess each group’s level of readiness to change and coach them to develop short-term goals for their CE activities.

### Data analysis

Descriptive statistics were used to summarize demographic data and the data collected by the FACE measure. Quantitative psychometric analyses (e.g., internal consistency) were not considered appropriate for the type of data collected. A case example is provided to illustrate how categorical frequency data and qualitative data from the FACE are shared with MBHI teams during follow-up meetings.

## Results

### Participants

Across eight initiatives, 28 MBHI key leaders and team members responded to the survey with an average of 3.5 respondents per group (*SD* = 2.78). The saturation of respondents was 53.85%. The majority were female (*n* = 27), and there was a split between licensed providers (*n* = 16) and administrative or research team members (*n* = 12). There were 10 participants from the psychology division, five from psychiatry, and seven from developmental pediatrics.

There were 28 participants in the follow-up conversations, 15 of whom were survey respondents. Most conversation participants were female (*n* = 27), and there were more administrative and research team members (*n* = 16) than licensed providers (*n* = 12). There were nine participants from psychology, seven from psychiatry, and two from developmental pediatrics. See Table 1 for detailed demographic information.

**Table 1.** Survey and meeting participant demographic information

Demographics	Survey Respondents (%)	Conversation Participants (%)	Participated in both survey and conversation (%)
Total Participants (*n*)	28 (100)	28 (100)	15 (100)
Male	1 (3.5)	1 (3.5)	1 (6.6)
Female	27 (96.5)	27 (96.5)	14 (93.3)
Role			
Licensed Provider	16 (57.2)	12 (42.8)	6 (40.0)
Administrative or Research Team Member	12 (42.8)	16 (57.2)	9 (60.0)
Department/Division			
Behavioral Medicine and Clinical Psychology	10 (35.7)	9 (32.2)	7 (46.7)
Child and Adolescent Psychiatry	5 (17.9)	7 (25.0)	3 (20.0)
Developmental and Behavioral Pediatrics	7 (25.0)	2 (7.1)	1 (6.6)
Other	6 (21.4)	10 (35.7)	4 (26.7)

*Note. n* = 28 survey participants. *n* = 28 conversation participants. *n* = 15 of the 28 participated in both.

### Survey results

Overall, survey results indicated that the MBHI initiative teams most frequently engage audiences at the Consult level. Specifically, groups have administered surveys, conducted group listening sessions, and given advisory council presentations. See Table 2 for aggregate results.

**Table 2.** Aggregated results of frequency of active community engagement (FACE) survey across initiatives

	Patients/Youth	Families/Caregivers	Community Members/Leaders
Inform	0.062	0.875	1.212
Consult	0.100	1.037	1.325
Involve	0.062	0.987	1.212
Collaborate	0.062	0.800	1.300
Shared Leadership	0.000	0.325	1.125
Average:	0.058	0.805	1.235

*Note.* Results were averaged across respondents (*n* = 28) from eight Mental and Behavioral Health Institute (MBHI) initiatives. Numbers represent frequency of engagement at each level from 0 (not engaged at all) to 4 (engaged several times per month).

The MBHI initiative teams reported most frequently engaging with the Community Members/Community Leaders population, followed by Caregivers. Youth and patients were the group the initiatives have been engaged with the least. With the Community Members/Community Leaders group, initiatives most often engaged community practice providers, schools, and non-profit community mental health resources.

### Case example of follow-up conversation

To demonstrate the process of how the FACE survey results directed the follow-up conversation, we will provide an example of the Family Navigation initiative. The Family Navigation team consisted of eight team members – five completed the FACE survey, seven participated in the conversation, and four participated in both. Table 3 shows an example of the survey results for this initiative, indicating the population, each team members’ rating, and the average score for each level of engagement. Overall, the results for the Family Navigation survey indicated that patients/youth are not engaged at any level (0.0), caregivers/families are engaged most often at the Inform level (1.2) followed closely by the Consult level (1.0), and community members/community leaders are not engaged (0.0).

**Table 3.** Family navigation survey quantitative and qualitative survey results

Level of Engagement	We are not engaged at this level currently (0 points)	We have engaged at this level in the past, but are not currently engaged (0 points)	We engage at this level 1-3 times per year (1 point)	We engage at this level quarterly (2 points)	We engage at this level monthly (3 points)	We engage at this level several times a month (4 points)	Average points across respondents
Inform	XX			XXX			1.2
Consult	X	X	X	XX			1.0
Involve	XX		XXX				0.6
Collaborate	XXX		X	X			0.6
Shared Leadership	XXXXX						0.0

**Qualitative Responses:**

Group Level Assessments (GLAs) at the start of the project to start us down the path, quarterly updates provided to those families.

Developing the role of family navigator.

Use of Caregiver Engagement during GLA sessions. Quarterly Email update on progress of program and future inclusion of Peer and Caregiver voice in Program Design.

Email follow up sent quarterly to GLA families on our progress to date; GLAs conducted in Spring/Summer 2024 with caregivers.

*Note. n* = 5 survey participants. GLA = Group level assessment.

The CE team met with the Family Navigation team and shared the results of the survey. The goal of the conversation was to develop a specific, measurable, attainable, relevant, and timely (SMART) goal for the short-term based on the survey results. In this meeting, the MBHI CE team worked with the Family Navigation team to gain buy-in by identifying barriers to increasing engagement and discussing program priorities and needs. The team identified time and team readiness as the top barriers. Based on their feedback, the lack of engagement with patients/youth, and the previous levels of engagement with caregivers/families, the team chose to focus on engaging both populations at the Collaborate level. They set the following SMART goal: “The Family Navigation team will engage 3–5 patients and/or caregivers at the Collaborate level by including them on the planning committee by July 2025.” The CE team held additional follow-up meetings with this team to support them in attaining their goal.

## Discussion

Patient perspectives and input have been increasingly recognized as essential to improving healthcare outcomes [10]. In the development of a new institute focused on pediatric mental and behavioral health, leadership determined the inclusion of patient, family, and community member voice to be of high priority at the outset and throughout implementation of key initiatives. Based on established CE models, the MBHI CE team developed a measure to describe the frequency of engagement and provide qualitative information to inform the creation of measurable CE goals. While the number of evaluation tools for community engagement has been growing in recent years, the team identified the need for a user-friendly measure that could provide a systematic way of measuring frequency of CE efforts over time and across initiatives. Since the MBHI is in early development of aligning three previously distinct healthcare divisions that encompass a multitude of programs, clinics, and research efforts, this tool not only provided an initial baseline of current CE efforts that had not yet been systematically collated but also established a shared language and framework to use with teams around CE through use of the IAP [2] model in our follow-up meetings. As this measure includes its foundation in CE theory and established CE models, it may be more readily reproducible to other programs or institutions.

Given that the key MBHI initiatives differ across clinical, research, and community outreach efforts, the FACE tool was effective for our team to have a single measure across teams. Additionally, this tool gave our team the ability to aggregate engagement data for the MBHI as a whole. Thus, our team could easily develop an overarching CE goal for the institution (e.g., increase frequency of engagement over the next 6 months) as well as identify specific targets for each individual initiative (e.g., achieve goals at *Involve* level of engagement with caregivers in 3 months).

A strength of the FACE tool is the ability to capture information about community partnerships with youth and patients, parents and caregivers, and community members (i.e., via survey and qualitative data) while providing an understanding of team’s goals and efforts. Lower participation among clinical leads in the conversations, compared to administrative and support staff, may have been due to their more limited availability. Even still, the evaluation approach is intended to be inclusive and captured multiple perspectives of team members engaged in CE activities.

Other established CE measures have utilized a continuum for measurement. For example, Clark *et al* [11]. described policy and system-level change related to the work of coalitions on asthma management as *peripheral*, *intermittent*, *ongoing*, or *core.* While the FACE tool is similar in that the continuum of CE efforts are measured, the FACE survey is simplified to reduce the possibility of judgment-based responses. Additionally, the tool can be used by both healthcare teams and community or patient partners to quantify the frequency of engagement and provide a measure of interobserver agreement.

### Limitations and future directions

Because the FACE tool is inherently limited to self-report of CE activities, it does not reflect specific clinical outcomes, research outcomes, or the engaged populations’ experiences. While assessing outcomes was not an immediate goal of this pilot stage of the measure, as the initiatives begin making progress, tracking changes to outcomes of interest to the MBHI (e.g., referrals, scheduled visits, billed hours) will reflect tangible outcomes of CE. The community’s experience of engaging with the MBHI is also a pertinent outcome and a way to measure the MBHI’s fidelity to the tenants of the IAP [2] model.

The FACE tool is also limited by the respondents’ understanding of the CE model. The survey included background information and definitions of each level with examples; however, the concepts were new for some of the survey participants. Education about CE and how various activities are categorized was an important function of the follow-up meetings, as the CE team further explained the model and how to most accurately reflect upon survey items. Therefore, it is expected that future waves of data collection will be more precise as the institution at large comes to a unified understanding of the CE continuum and how to define and describe their CE activities. Because these definitions of CE are relatively new to the institute at large, we did not include more specific measures of effort involved in specific CE activities at different levels. A next step is to understand time and scope of effort at the ground level to define in practical terms how engagement works within teams.

Similar to the limitations of other CE measures, our measure has not yet undergone full psychometric testing. As the measure is in early stages of piloting and these steps were not considered appropriate at this time, this type of testing is an important future direction to ensure that the measure has appropriate reliability and validity. Specifically, we will be able to assess construct validity as we begin to collect community-facing measures (i.e., convergent validity) of CE and outcomes associated with CE (i.e., hypothesis testing). Notably, the FACE is the only measure of CE frequency at five levels of engagement activities (e.g., inform to shared leadership), and this approach lends itself to face validity and construct validity.

## Conclusions

In sum, the MBHI CE team developed the FACE tool, a measure of CE that is user-friendly and based on established models, making it highly reproducible. The FACE measures engagement across initiatives and frequency, with the ability to understand activities and track progress over time. This measure may be especially useful for academic institutions and universities aiming to effectively engage youth, families, and communities.

## Supporting information

10.1017/cts.2025.10145.sm001 Murphy et al. supplementary material Murphy et al. supplementary material

## Supplementary material

The supplementary material for this article can be found at https://doi.org/10.1017/cts.2025.10145.
