# Supplementary material for: Rulers of engagement: A novel approach to measuring engagement in a large Mental and Behavioral Health Institute
Source: J Clin Transl Sci. 2025 Sep 2;9(1):e222. doi: 10.1017/cts.2025.10145 (PMC12529633; doi:10.1017/cts.2025.10145)
Supplement: Murphy et al. supplementary material [file S2059866125101453sup001.pdf]

MBHI Community Engagement Survey

* Required

* This form will record your name, please fill your name.

1. First and Last Name *

2. Please select which MBHI Initiative/Project you are involved with: *

If you are involved in more than one MBHI initiative, please complete a separate form for each.

- ◯ Evidence-Based Certificate Program
- ◯ Exposure Coaching
- ◯ Family Navigation
- ◯ Fisher Center
- ◯ Front Door
- ◯ Integrated Behavioral Health - Community
- ◯ Integrated Behavioral Health - Hospital
- ◯ Interventional Psychiatry
- ◯ Mayerson Center
- ◯ PINQ Learning Network
- ◯ Project ECHO
- ◯ School Strategy
- ◯ Specialized Care Pathways
- ◯ Zero Suicide
- ◯ Other

3. Please select to what degree your initiative engages **youth and/or patients** at each level of involvement: *

Youth/patients *could include youth within CCHMC, the community, and schools under the age of 25.*

	We are not engaged at this level at this time	We have engaged at this level in the past, but are not currently engaged	We engage at this level 1-3 times per year	We engage at this level quarterly	We engage at this level monthly	We engage at this level several times a month
Inform	◯	◯	◯	◯	◯	◯
Consult	◯	◯	◯	◯	◯	◯
Involve	◯	◯	◯	◯	◯	◯
Collaborate	◯	◯	◯	◯	◯	◯
Shared Leadership	◯	◯	◯	◯	◯	◯

4. Briefly provide some examples of what you're currently doing to engage **youth and/or patients**:

5. Please select to what degree your initiative engages **caregivers and/or families** at each level of involvement: *

Families/Caregivers *could include CCHMC patient families and caregivers and caregivers of youth in the community.*

	We are not engaged at this level at this time	We have engaged at this level in the past, but are not currently engaged	We engage at this level 1-3 times per year	We engage at this level quarterly	We engage at this level monthly	We engage at this level several times a month
Inform	◯	◯	◯	◯	◯	◯
Consult	◯	◯	◯	◯	◯	◯
Involve	◯	◯	◯	◯	◯	◯
Collaborate	◯	◯	◯	◯	◯	◯
Shared Leadership	◯	◯	◯	◯	◯	◯

6. Briefly provide some examples of what you're currently doing to engage **families and/or caregivers:**

7. Please select to what degree your initiative engages **community residents and/or community leaders** at each level of involvement: *

__Community residents/leaders__ could include community members, non-CCHMC professionals, and community organization leaders and members.

	We are not engaged at this level at this time	We have engaged at this level in the past, but are not currently engaged	We engage at this level 1-3 times per year	We engage at this level quarterly	We engage at this level monthly	We engage at this level several times a month
Inform	◯	◯	◯	◯	◯	◯
Consult	◯	◯	◯	◯	◯	◯
Involve	◯	◯	◯	◯	◯	◯
Collaborate	◯	◯	◯	◯	◯	◯
Shared Leadership	◯	◯	◯	◯	◯	◯

8. Briefly provide some examples of what you're currently doing to engage **community residents and/or community leaders:**

9. Please share any other stakeholder groups that you engage:

10. Please select any barriers you see to increasing your engagement with youth/patients, families/caregivers, and community residents/leaders. *

- ☐ Time
- ☐ Money
- ☐ Lack of support
- ☐ Small patient population
- ☐ Team readiness
- ☐ Other

This content is neither created nor endorsed by Microsoft. The data you submit will be sent to the form owner.

Microsoft Forms
